# Role of EPO and TCF7L2 Gene Polymorphism Contribution to the Occurrence of Diabetic Retinopathy

**DOI:** 10.1155/2022/6900660

**Published:** 2022-05-29

**Authors:** Chao Liu, Ga-Li Bai, Ping Liu, Lin Wang

**Affiliations:** ^1^Department of Ophthalmology, The Fourth Affiliated Hospital of Harbin Medical University, Harbin, 150001 Heilongjiang Province, China; ^2^Department of Ophthalmology, The First Affiliated Hospital of Harbin Medical University, Harbin, 150001 Heilongjiang Province, China

## Abstract

*Objective*: For studying the association of EPO (rs551238), EPO (rs1617640), and TCF7L2 (rs7903146) gene with diabetic retinopathy in Northern Chinese population. *Methods*: We conducted a case-control study, which enrolled 680 subjects and performed SNP genotyping and calculated allele frequencies. *Results*: When comparison was performed between DR patients and normal persons, the EPO (rs551238) AA genotype has a significant risk association with DR, and AC genotype has a significant protective association with DR. The EPO (rs551238) A allele has a significant risk association with DR, and C allele has a significant protective association with DR. When comparison was performed between DR patients and DM patients, the EPO (rs551238) CC genotype has a significant protective association with DR; the EPO (rs551238) A allele has a significant risk association with DR; and C allele has a significant protective association with DR. When comparison was performed between DR patients and normal persons, the EPO (rs1617640) GT genotype has a significant protective association with DR, and TT genotype has a significant risk association with DR. The EPO (rs1617640) G allele has a significant protective association with DR, and T allele has a significant risk association with DR. In addition, we found that TT genotype does not exist in rs7903146 of TCF7L2 in Chinese population so that the data could not be used. *Conclusions*: EPO (rs551238, rs1617640) genotype is a susceptible gene for DR in Chinese type 2 diabetic patients, especially the high-risk PDR.

## 1. Introduction

It is well known that the main complication of diabetes mellitus is diabetic retinopathy. The main reasons for loss of vision are complications of proliferative diabetic retinopathy (PDR) and diabetic maculopathy resulted by vitreous hemorrhage, tractional retinal detachment, and neovascular glaucoma. It is reported that compared to 2010, the number of patients with diabetes will increase 69% in developing countries and 20% in industrialized countries by 2030 [1].

Although most patients can not notice early retinal changes, over time, potentially vision-threatening retinal changes develop in patients with diabetes [2]. Diabetic retinopathy is the main microvascular complication of diabetes retinopathy, and 3%-4% of European are affected [3, 4]. In industrialized nations, diabetes mellitus is the main cause of blindness in working-age population [3]. There is about one-third of patients that have characteristics of diabetic retinopathy once diabetes mellitus is surely diagnosed.

It is reported that, in patients with type 1 diabetes, proliferative diabetic retinopathy is an independent marker of long-term nephropathy [5]. The incidence rate of DR is 25.2% in Chinese DM patients, and the ratio is 12.4% in patients who were primary diagnosed as type 2 DM. With the trend of younger diabetic patients in recent years, diabetic nephropathy and other complications have been effectively controlled. DR is the most common blind disease that affects the quality of life of diabetic patients. How to prevent and treat DR has always been a concern of people [3].

It is reported that EPO has the relationship with ischemic retinal disease such as PDR (7.8). The concentration of EPO in tissues is regulated by hypoxia, and it is a multifunctional glucoprotein [6, 7]. The function of EPO is stimulating the formation of red blood cells and increasing their differentiation of erythrocyte precursors [8]. It is said that hypoxia is the main stimulus factor which regulates the role of erythrocyte [9, 10]. The EPO and its receptors are produced by a para-endocrine system in retina. It is suggested that EPO prevents neurons from apoptosis resulted by ischemic damage [10]. EPO also plays a role of stimulating the differentiation and migration of endothelial cells [11]. The EPO concentration in patients suffered from PDR was significantly higher than those normal persons [12]. There is a paper [12] reported that EPO is able to stimulate retinal angiogenesis in PDR, and EPO is activated locally in the retina.

Transcription factor 7-like2 (TCF7L2) belongs to T-cell-specific high-mobility group. It is reported that single-nucleotide polymorphism(SNP) in human TCF7L2 genes has a close relationship with occurrences of type 2 diabetes after extensive genome studies in numerous ethnic populations [13–15]. TCF7L2 single nucleotide polymorphisms lead to decreased *β*-cell function, including impaired insulin secretion and processing, and increased insulin resistance [16, 17]. However, the mechanism of TCF7L2 gene function and how its genetic polymorphism influence the susceptibility of type 2 diabetes has not been elucidated. There are several reports suggested that the expression of TCF7L2 is up-regulated in diabetic subjects [18].

In this study, we aimed to analyze the association of EPO (rs551238), EPO (rs1617640), and TCF7L2 (rs7903146) gene with DR in Northern Chinese population.

## 2. Methods

We strictly complied with the Declaration of Helsinki when performing the study, and all participants signed the informed consent forms. The research was approved by the Ethics Committee of the Eye Hospital of the First Clinical Hospital Affiliated Harbin Medical University, Harbin, China (No. HMU7703). The subjects were 320 Han Chinese patients with DR, 235 normal healthy controls, and 125 Han Chinese patients with DM (without DR). All selected subjects were patients who attended the Eye Hospital of the First Clinical Hospital Affiliated Harbin Medical University, Harbin, China, from April 2014 to January 2015.

All tested subjects are all Hei Longjiang Han Chinese people, and there are no blood relationship among the tested subjects. We strictly conform to American DM diagnosed standard and ensure the ages of all tested subjects are above or equal to 30 years old; courses of the DM are above or equal to 5 years. We detailed record the patients materials and medical history which contained age, gender, body length, body weight, condition of cigarette smoking and alcohol drinking, and course of the disease. We also measured the systolic pressure and diastolic pressure of patients.

All enrolled study cases all performed strict ophthalmology examination contained vision, slit lamp examination of anterior segment, and intraocular pressure. Fundus examination was performed by two skilled physicians using an ophthalmoscope after pupil dilation with Mydrin. If there was not pathology found in eyeground (microaneurysm, retinal hemorrhage, cotton-wool spot, formation of retinal neovascularization, vitreous hemorrhage), these patients will belong to DR group. Fundus fluorescein angiography (FFA) was performed in all patients diagnosed with DR. The eyeground condition of patients was considered at different stages in relation to DR classification criteria by two skillful doctors.

We also chose unrelated healthy subjects who were selected from the Health Examination Department of our hospital as ARC healthy controls. All patients and healthy controls were sex-, age-, and ethnically matched.

DR, diabetes, cataract, glaucoma, fundus oculi disease, hypertension, tumor and other related ocular diseases were not observed in the healthy control group. We performed full ophthalmic examination including visual acuity examination, slit lamp biomicroscope examination, lens examination, and fundus examination in all DR, DM patients, and healthy control subjects. Blood sampling and DNA extraction were also carried out.

Venous blood (2 ml) was collected in EDTA tubes from all DR, DM patients, and healthy controls. Genomic DNA was extracted from the collected blood using an Omega DNA blood extraction kit (Omega, USA) according to the manufacturer's instructions. All blood samples were stored in 1.5-ml Eppendorf tubes and then centrifuged at -20°C.

During SNP genotyping, based on earlier studies which suggested that EPO and TCF7L2 polymorphisms were related to DR and other age-related diseases, we chose EPO (rs551238), EPO (rs1617640), and TCF7L2 (rs7903146) as candidate SNPs. Amplification of the target DNA in EPO and TCF7L2 is analyzed by LDR assay using the primers as shown in Table 1. The EPO and TCF7L2 reactions were performed in a 15 *μ*l reaction mixture containing 1 *μ*l genomic DNA, 0.3 *μ*l Taq enzymes (Fermentas Corporation, EP 0460), 0.15 *μ*l primer mixture, 0.3 *μ*l dNTPs (Fermentas Corporation, R0192), 1.5 *μ*l MgCl_2_, and 1.5 *μ*l 10 × buffer for amplification of DNA. The conditions were as follows: initial denaturation at 94°C for 3 min followed by 35 cycles of denaturation at 94°C for 15 s, annealing at 62°C for 15 s, extension at 72°C for 30 s, and a final extension at 72°C for 3 min.

The ligation detection reaction (LDR) was performed in a 10 *μ*l reaction mixture containing 3 *μ*l PCR reaction product, 1 *μ*l 10 × Taq DNA ligase buffer, 0.125 *μ*l Taq DNA ligase (40 *μ*/*μ*l, NEB Corporation M02081), and 0.01 *μ*l probe (10p per strip). This was followed by 30 cycles at 94°C for 30 s and 60°C for 3 min. 1 *μ*l extension products was obtained, and 8 *μ*l loading sample was added. After denaturation at 95°C for 3 min, the mixture was immediately placed in an ice bath, and then, a sequencer (ABI 3730XL) was used. The probe sequences of the EPO and TCF7L2 genes are shown in Table 2.

Statistical analysis: In this study, the Chi-square test was used to assess the Hardy-Weinberg equilibrium. We directly counted the number of genotypes and alleles. SPSS (version 17.0, SPSS Inc., Chicago, IL, USA) was used to compare allele and genotype frequencies among DR, DM patients, and healthy controls. The comparison of genotypes and the frequencies of alleles was analyzed by *χ*^2^ test. Meanwhile, OR value and 95% confidence area (95% CI) were calculated. *P* value <0.05 was considered statistically significant.

## 3. Results

**Figure 1:**
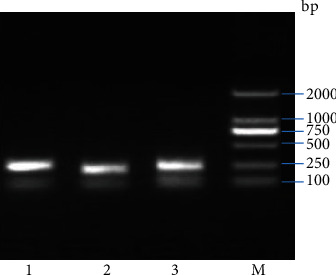
Ethidium bromide stained 1% agarose gel of products from PCR reactions using rs551238 primers, rs1617640 primers, and rs7903146 primers. Lanes 1 to 3: products from reactions with primers for rs551238, rs1617640, and rs7903146. Lane 4: molecular size standards.

The results showed that the EPO and TCF7L2 genetic variants were in Hardy-Weinberg equilibrium both in DR patients and in the healthy controls. The PCR reaction products of EPO (rs551238), EPO (rs1617640), TCF7L2 (rs7903146) in ethidium bromide were stained with 1% agarose gel. The result is shown in Figure 1.

The primers used for LDR analysis of the EPO and TCF7L genes and probe sequences of the EPO and TCF7L genes are, respectively, shown in Table 1 and Table 2.

When comparison was performed between DR patients and normal persons, the EPO (rs551238) AA genotype has significant risk association with DR susceptibility with OR of 1.960 (1.374-2.795) (*P* = 0); AC genotype has a significant protective association with DR susceptibility with OR of 0.517 (0.360-0.742) (*P* = 0.001). The EPO (rs551238) A allele has a significant risk association with DR susceptibility with OR of 1.690 (1.248-2.288) (*P* = 0.001); C allele has a significant protective association with DR susceptibility with OR of 0.592 (0.437-0.801) (*P* = 0.001). The Bonferroni correction was performed (*P* = 0.001 < 0.05). The results are shown in Table 3.

When comparison was performed between DR patients and DM patients, the EPO (rs551238) CC genotype has a significant protective association with DR susceptibility with OR of 0.375 (0.138-1.022) (*P* = 0.047). The EPO (rs551238) A allele has a significant risk association with DR susceptibility with OR of 1.524 (1.052-2.206) (*P* = 0.025). C allele has a significant protective association with DR susceptibility with OR of 0.656 (0.453-0.950) (*P* = 0.025). The Bonferroni correction was performed (*P* = 0.032 < 0.05). The results are shown in Table 4.

When comparison was performed between DR patients and normal persons, the EPO (rs1617640) GT genotype has a significant protective association with DR susceptibility with OR of 0.649 (0.451-0.933) (*P* = 0.019); TT genotype has a significant risk association with DR susceptibility with OR of 1.594 (1.116-2.278) (*P* = 0.010). The EPO (rs1617640) G allele has a significant protective association with DR susceptibility with OR of 0.678 (0.498-0.923) (*P* = 0.013); T allele has a significant risk association with DR susceptibility with OR of 1.476 (1.084-2.010) (*P* = 0.013). The Bonferroni correction was performed (*P* = 0.013 < 0.05). The results are shown in Table 3.

When comparison was performed between DR patients and DM patients, there were no obvious differences in the EPO (rs1617640). The results are shown in Table 4.

In our study, we found TT genotype did not exist in rs7903146 of TCF7L2 in Chinese population so the data could not be used.

There was no obvious significance between normal persons and DM patients, and the table are shown in Table 5.

## 4. Discussion

We performed a case-control study of the three polymorphisms associated with DR in Heilongjiang Han Chinese populations. Our results showed that the SNP, EPO1 (rs551238), and EPO2 (rs1617640) were significantly associated with susceptibility to DR.

In response to anemia and hypoxia (26), EPO, which is a glycoprotein hormone, plays an important role in increasing the production of red blood cells, and EPO is produced in adult kidney and fetal liver.

Watanabe et al. suggested that EPO is an ischemia-induced angiogenic factor in both patient and animal models with PDR [12]. It is reported by Garcia-Ramirez et al. [19] that compared with nondiabetic controls, EPO is overexpressed in the retina of DM patients. Tong et al. [20] reported that rs1617640 in the expression of EPO play an important role in the occurrence of DM. However, about the relationship of rs551238, rs1617640, and DR, the related materials are still not sufficient. In our study, we dedicated to study the relationship among rs551238 and rs1617640 and the occurrence of DM and call into question the validity of association between rs551238, rs1617640, and DR.

The role of transcription factor 7-like 2(TCF7L2) is regulating fundamental processes such as growth of vascular and mediating pathological neovascularization in DM. In addition, TCF7L2 has an important role in the Wnt-signaling pathway [21]. There also is a study indicated that TCF7L2 gene has close relationship with macrovascular and microvascular complications [22]. There were many studies indicated that rs7903146 in TCF7L2 has a close relationship with type 2 DM [13, 23]. However, the mechanism of type 2 DM still remains unclear, and the association between TCF7L2 and DR has been conflicting [13, 22]. Here, we studied the role of TCF7L2 (rs7903146) in DR using a genetic association research.

The product of TCF7L2 is a transcription factor which has the function of activating genes downstream in Wnt signaling pathway of type 2 DR [24–27]. About how the TCF7L2 influence the susceptibility of DR needed to be elucidated. The results of our study show that the *P* (*P* = 0) value of the AA genotype of EPO1 (rs551238) was significantly increased, while the *P* (*P* = 0) value of AC genotype of EPO1 (rs551238) was significantly decreased compared with controls. Our study also show that the *p* (*p* = 0.001) value of the “A” allele of EPO1 (rs551238) was significantly increased, while the *p* (*p* = 0.001) value of “C” allele of EPO1 (rs551238) was significantly decreased compared with controls. When comparison was performed between DR and DM patients, there were no obvious differences in genotype of AA, AC, and CC. The results still suggested that the *P* (*P* = 0.025) value of the “A” allele of EPO1 (rs551238) was significantly increased, while the *p* (*p* = 0.025) value of “C” allele of EPO1 (rs551238) was significantly decreased when comparison was performed between DR patients and DM patients.

The results of our study showed that the *p* (*p* = 0.019) value of GT genotype of EPO2 (rs1617640) was significantly decreased compared with control. Our study also show that the *p* (*p* = 0.010) value of TT genotype of EPO2 (rs1617640) was significantly increased compared with controls. Our study also show that the *p* (*p* = 0.013) value of the “G” allele of EPO2 (rs1617640) was significantly decreased, while the *p* (*p* = 0.013) value of “T” allele of EPO2 (rs1617640) was significantly increased. When comparison was performed between DR patients and DM patients, there are no obvious differences in genotype of :GG GT TT" and allele of “G” and “T.”

When we studied the differences in rs7903146 among DR patients, DR patients, and normal persons, we found that “TT” genotype did not existed in Heilongjiang Chinese people. So the data studied TCF7L2 (rs7903146) was not balanced, and the data cannot elucidate the role of TCF7L2 (rs7903146) in occurrence of DR.

When comparison was performed between DM patients and normal persons, there are no obvious differences which suggested that EPO1 (rs551238), EPO2 (rs1617640), and TCF7L2 (rs7903146) did not play role in occurrence of DM, and the three SNPs are not signs of DM.

From our study, we found that allele “A” was the risk factor and allele “C” was the protective factor when compared between DR patients and normals, diabetics and normals in EPO1 (rs551238 ).

Our data showed that “G” allele plays a protective role, while “T” allele plays a risk role in occurrence of DR. However, when comparison was performed in DR and DM patients, there were no obvious differences existed in genotypes and alleles. We can see when a patient affected from DM, the “T” allele is not a risk factor for distinguishing DR and DM, but the “T” allele is really a significant sign for distinguishing DR patients and normal people. So we can concluded that if “T” allele is the main allele in a DM patient, we could not predict that he or she will suffer from DR, but at the least, the “T” allele is a risk signal for the DM patient, because it is a significant risk factor when comparison was performed between DR patients and normal people.

From our data, it can be seen that the TT genotype does not exist in the Han people of Heilongjiang, China, so the data is unbalanced. We can accurately determine which genotypes and alleles play a risk factor in the occurrence of DR, or we need a large number of samples to determine the role of TCF7L2 (rs7903146) in DR development.

To decrease the influencing factors in our research, we adopted measures verifying the study findings. We selected patients with DR and if complicated by other systemic or ophthalmology complications, we will exclude these patients. We adopted LDR assay to determine the genotype.

Similar to other gene polymorphism research, our study had some limitations. Firstly, the biological function of SNP, EPO1 (rs551238), EPO2 (rs1617640), and TCF7L2 (rs7903146) requires further investigation. Secondly, the size of the patient group in our study was relatively small, and the number of patient-control sample will influence the susceptibility of gene detection. In addition, we only recruited patients from the Han Chinese people, and other ethnic groups should be recruited in our future study. The results required in our study should perform a larger sample size which included other ethnic groups. Thirdly, a stratification analysis on the relationship between different ages of DR and the four gene polymorphisms should have been performed. Moreover, we calculated the odds ratio of different genotypes and alleles of the studied SNPs. The reference genotype or allele for each SNP for calculating the odds ratio should be analyzed in future investigations.

Overall, our study identify “A” allele of EPO1 (rs551238) as a risk factor and “T” allele of EPO2 (rs1617640) as a risk signal in occurrence of DR. In future studies, the biological functions of EPO1 (rs551238) and EPO2 (rs1617640), contributing to the occurrence of DR, should be evaluated. To our knowledge, the present study is the first report of an association among EPO1 (rs551238), EPO2 (rs1617640), and DR in a Han Chinese population. Meanwhile, it has been reported that EPO is associated with DR by regulating caspase-3 expression and oxidative stress [28]. EPO regulates the inner blood-retinal barrier in DR through repressing microglia phagocytosis by Src/Akt signaling [29]. The potential mechanism between the genotype of EPO with the risk of DR should be confirmed in future investigations. Moreover, the investigations of the regulatory mechanisms underlying TCF7L2 are still limited, and the mechanism between the genotype of TCF7L2 with the risk of DR should be explored in the future.

Taken together, our finding demonstrated the independent contribution of EPO gene polymorphism to DR susceptibility in the Heilongjiang Han Chinese population. We hope that our study will establish background data for further investigation into the mechanism of EPO genes and the development of DR.

## Figures and Tables

**Table 1:** Primers used for LDR analysis of the EPO and TCF7L genes.

Rs number	Primers	Amplification bands
rs551238	CCAGGGTTGGCAGCTGTTACC	227 bp
	TCTCACACAGCCTGTCTGACC	

rs1617640	AGCTAGGCTGCATTGCTGAGT	219 bp
	CACCCATTTGACAGATGAGGA	

rs7903146	TGCCTCAAAACCTAGCACAGCT	229 bp
	GTAGCAGTGAAGTGCCCAAGC	

**Table 2:** Probe sequences of the EPO and TCF7L genes.

Rs number	Probe sequences
rs551238	CACCTTATTGACCAGCGTAGGCAGA-FAM-
rs1617640	GAGTGAGATTCCCAGAGCAGGAGACTTT-FAM-
rs7903146	TATATAATTTAATTGCCGTATGAGGCACTTT-FAM-

**Table 3:** Frequences of genotypes and alleles of EPO and TCF7L genes polymorphisms in diabetes retinopathy patients and normal controls.

*SNP*	*Genotype DR (%)*	*Normal controls (%)*		*pvalue*	*OR (95% CI)*
	Allele	(*n* = 320)			(*n* = 235)
	AA	(71.88%) 230	(56.60%) 133	≤0.001	1.960 (1.374-2.795)
	AC	(25.63%) 82	(40.00%) 94	≤0.001	0.517 (0.360-0.742)

rs551238 (EPO1)	CC	(2.50%) 8	(3.40%) 8	0.529	0.728 (0.269-1.967)
	A	(97.50%) 312	(96.60%) 227	0.001	1.690 (1.248-2.288)
	C	(28.13.3%) 90	(47.66%) 112	0.001	0.592 (0.437-0.801)
	GG	(1.88%) 6	(2.98%) 7	0.396	0.622 (0.206-1.877)
	GT	(26.89%) 86	(36.17%) 85	0.019	0.649 (0.451-0.933)

rs1617640 (EPO2)	TT	(71.25%) 228	(60.85%) 143	0.01	1.594 (1.116-2.278)
	G	(28.75.3%) 92	(39.15%) 92	0.013	0.678 (0.498-0.923)
	T	(98.13%) 314	(97.02%) 228	0.013	1.476 (1.084-2.010)
	CC	(87.19%) 279	(94.89%) 223	0.002	0.366 (0.188-0.713)
	CT	(12.81%) 41	(5.11%) 12	0.002	2.731 (1.402-5.320)

rs7903146 (TCF7L2)	TT	(0.00) 0	(0.00) 0		
	C	(100.00%) 320	(100.00%) 235	0.003	0.383 (0.199-0.737)
	T	(12.81%) 41	(5.11%) 12	0.003	2.612 (1.357-5.028)

OR: odds ratio.

**Table 4:** Frequences of genotypes and alleles of EPO and TCF7L genes polymorphisms in diabetes retinopathy patients and diabetes mellitus patients.

*SNP*	*Genotype*	*DR (%)*	*DM (%)*	*p* *value*	*OR (95% CI)*
	Allele	(*n* = 320)			(*n* = 125)
	AA	(71.88%) 230	(63.20%) 79	0.074	1.488 (0.961-2.305)
	AC	(25.62%) 82	(30.40%) 38	0.308	0.789 (0.500-1.245)

rs551238 (EPO1)	CC	(2.50%) 8	(6.40%) 8	0.047	0.375 (0.138-1.022)
	A	(97.50%) 312	(78.4%) 196	0.025	1.524 (1.052-2.206)
	C	(29.13%) 90	(36.80%) 46	0.025	0.656 (0.453-0.950)
	GG	(1.88%) 6	(3.20%) 4	0.397	0.578 (0.160-2.084)
	GT	(26.88%) 86	(29.60%) 37	0.563	0.874 (0.554-1.380)

rs1617640 (EPO 2)	TT	(71.25%) 228	(67.20%) 84	0.402	1.210 (0.775-1.888)
	G	(28.75%) 92	(32.80%) 41	0.326	0.824 (0.559-1.214)
	T	(98.13%) 314	(96.80%) 121	0.326	1.214 (0.824-1.789)
	CC	(87.19%) 279	(96.00%) 120	0.006	0.284 (0.109-0.735)
	CT	(12.81%) 41	(4.00%) 5	0.006	3.527 (1.360-9.145)

rs7903146 (TCF7L2)	TT	(0) 0	(0) 0		
	C	(100.00%) 320	(100.00%) 120	0.008	0.298 (0.116-0.763)
	T	(12.81%) 41	(4.00%) 5	0.008	3.354 (1.310-8.588)

OR: odds ratio.

**Table 5:** Frequences of genotypes and alleles of EPO and TCF7L genes polymorphisms in normal controls and diabetes mellitus patients.

*SNP*	*Genotype*	*Normal controls (%)*	*DM (%)*	*pvalue*	*OR (95% CI)*
	Allele	(*n* = 235)			(*n* = 125)
	AA	(56.60%) 133	(63.20%) 79	0.225	0.759 (0.486-1.186)
	AC	(40.00%) 94	(30.40%) 38	0.072	1.526 (0.962-2.422)

rs551238 (EPO1)	CC	(3.4%) 8	(6.40%) 8	0.189	0.515 (0.189-1.408)
	A	(95.60%) 227	(93.60%) 117	0.583	0.902 (0.623-1.304)
	C	(47.66.4%) 112	(36.80%) 46	0.583	1.109 (0.767-1.604)
	GG	(2.98%) 7	(3.20%) 4	0.908	0.929 (0.627-3.235)
	GT	(36.17%) 85	(29.60%) 37	0.148	1.409 (0.885-2.244)

rs1617640 (EPO 2)	TT	(60.85%) 143	(67.20%) 84	0.235	0.759 (0.481-1.197)
	G	(39.15%) 92	(32.80%) 41	0.328	1.216 (0.822-1.798)
	T	(97.02%) 228	(96.80%) 121	0.328	0.823 (0.556-1.217)
	CC	(94.89%) 223	(96.00%) 120	0.638	0.774 (0.266-2.250)
	CT	(5.11%) 12	(4.00%) 5	0.638	1.291 (0.444-3.725)

rs7903146 (TCF7L2)	TT	(0) 0	(0) 0		
	C	(100.00%) 235	(100.00%) 125	0.642	0.779 (0.271-2.236)
	T	(5.11%) 12	(4.00%) 5	0.642	1.284 (0.447-3.686)

## Data Availability

The datasets used during the present study are available from the corresponding author upon reasonable request.
